# Adherent-Invasive and Non-Invasive *Escherichia coli* Isolates Differ in Their Effects on *Caenorhabditis elegans’* Lifespan

**DOI:** 10.3390/microorganisms9091823

**Published:** 2021-08-27

**Authors:** Maria Beatriz de Sousa Figueiredo, Elizabeth Pradel, Fanny George, Séverine Mahieux, Isabelle Houcke, Muriel Pottier, Chantal Fradin, Christel Neut, Catherine Daniel, Antonino Bongiovanni, Benoît Foligné, Marie Titécat

**Affiliations:** 1 Univ. Lille, Inserm, CHU Lille, U1286-INFINITE-Institute for Translational Research in Inflammation, F-59000 Lille, France; mariabeatriz.desousafigueiredo@univ-lille.fr (M.B.d.S.F.); elizabeth.pradel@inserm.fr (E.P.); fanny.george@univ-lille.fr (F.G.); severine.mahieux@univ-lille.fr (S.M.); isabelle.houcke@univ-lille.fr (I.H.); muriel.pottier@univ-lille.fr (M.P.); christel.neut@univ-lille.fr (C.N.); 2 Univ. Lille, CNRS, Inserm, CHU Lille, Institut Pasteur de Lille, U1019-UMR 9017-Center for Infection and Immunity of Lille, F-59000 Lille, France; catherine.daniel@ibl.cnrs.fr; 3 Univ. Lille, Inserm, Institut Pasteur de Lille, U1167-RID-AGE, F-59000 Lille, France; chantal.fradin@univ-lille.fr; 4 Univ. Lille, CNRS, Inserm, CHU Lille, Institut Pasteur de Lille, US 41-UMS 2014-PLBS, F-59000 Lille, France; antonino.bongiovanni@univ-lille.fr

**Keywords:** adherent-invasive *E. coli* (AIEC), *Caenorhabditis elegans*, gut inflammation, inflammatory bowel disease (IBD), intestinal epithelial cell

## Abstract

The adherent-invasive *Escherichia coli* (AIEC) pathotype has been implicated in the pathogenesis of inflammatory bowel diseases in general and in Crohn’s disease (CD) in particular. AIEC strains are primarily characterized by their ability to adhere to and invade intestinal epithelial cells. However, the genetic and phenotypic features of AIEC isolates vary greatly as a function of the strain’s clonality, host factors, and the gut microenvironment. It is thus essential to identify the determinants of AIEC pathogenicity and understand their role in intestinal epithelial barrier dysfunction and inflammation. We reasoned that soil nematode *Caenorhabditis elegans* (a simple but powerful model of host-bacterium interactions) could be used to study the virulence of AIEC vs. non- AIEC *E. coli* strains. Indeed, we found that the colonization of *C. elegans* (strain N2) by *E. coli* impacted survival in a strain-specific manner. Moreover, the AIEC strains’ ability to invade cells in vitro was linked to the median lifespan in *C. elegans* (strain PX627). However, neither the *E. coli* intrinsic invasiveness (i.e., the fact for an individual strain to be characterized as invasive or not) nor AIEC’s virulence levels (i.e., the intensity of invasion, established in % from the infectious inoculum) in intestinal epithelial cells was correlated with *C. elegans*’ lifespan in the killing assay. Nevertheless, AIEC longevity of *C. elegans* might be a relevant model for screening anti-adhesion drugs and anti-invasive probiotics.

## 1. Introduction

Inflammatory bowel diseases (IBDs, including Crohn’s disease (CD) and ulcerative colitis (UC)) are caused by intricate genetic and environmental factors that alter intestinal homeostasis [1,2]. This inflammatory contexts results in dysbiosis [3], which is characterized by specific changes in the symbiont/pathobiont ratio of the gut microbiota [4]. Along with a significant reduction in overall microbial diversity, microbial imbalance leads to a fall in the abundance of beneficial bacteria and an increase in the abundance of potentially pathogenic bacteria. The latter include certain mucosa-associated bacteria (such as the Enterobacterales [5]) that might trigger or exacerbate IBDs in susceptible individuals.

Adherent-invasive *Escherichia coli* (AIEC) was originally identified as a specific pathogenic group in patients with ileitis developed CD in France [6,7]. Over the past two decades, research has shown that the AIEC pathotype is involved in the pathogenesis of IBD [8]; these bacteria are overrepresented in mucosal samples from patients with CD (relative to healthy controls) [9]. AIEC have also been evidenced in inflammatory diseases in animal [10] and appear to have a role in experimental models of colitis [11,12]. Moreover, the high prevalence of AIEC strains in patients with UC and CD suggests that the bacterium’s association with IBD in general is stronger than first anticipated [13,14]. AIEC are also abnormally abundant in individuals with colorectal cancer [15,16], patients with functional intestinal disorders, and asymptomatic people undergoing surveillance colonoscopy [17,18]. The significance of AIEC in healthy people (albeit at a lower frequency than in patients with IBDs) has yet to be determined; the presence of these strains might indicate a predysbiotic state (favoring infection), or an elevated risk of IBD, or other diseases involving a gut microbial imbalance. Surprisingly, the occurrence and abundance of intestinal AIECs in people with metabolic diseases and other immune disorders associated with low-grade inflammation have not been extensively studied. For example, it has not yet been determined whether AIECs are abnormally abundant in individuals with arthritis or whether the strains’ abundance is linked to the aging process [19]. *E. coli* is a versatile bacterial species that encompasses probiotic strains with beneficial properties, harmless commensal strains, and pathogenic strains found in the gastrointestinal tract in humans and animals. In fact, AIEC pathobionts might belong to the “gray zone” of commensal bacteria with pro-inflammatory potential and a propensity to disrupt intestinal epithelial homeostasis. This disruption might lead to a state of chronic inflammation via continuous stimulation of the mucosal immune system. It is therefore essential to understand the determinants of the AIEC’s pathogenicity within the host.

The AIEC phenotype is characterized by the ability to (i) adhere to and invade intestinal epithelial cells [6,20,21], (ii) survive and replicate within the macrophage phagolysosome, and (iii) trigger the release of pro-inflammatory cytokines. Otherwise, the AIECs are highly diverse in both genetic and phenotypic terms: new models tracking the within-host evolution of AIECs suggest that the strains stratify themselves into distinct subpopulations in vivo [22]. Depending on the host environment, the AIEC’s virulent phenotype may exert distinct degrees of pathogenicity via various metabolic, immune, and virulence factors [8,12,23]. Many virulence genes and factors have been described in AIEC pathobionts, and comparative genomics studies have evidenced a high degree of variability (including single nucleotide polymorphisms). Hence, the genetic-based identification of AIEC is challenging (for a review, see [8]). Although most AIEC strains are assigned to the B2 phylogroup, others are assigned to the A, B1, and D phylogroups. Hence, no potential genetic markers nor immunoreactivity appear to be specific and distributed across all AIEC strains and might be predictive of the phenotype explaining the lack of molecular tools for their rapid identification [24,25].

Nevertheless, the AIEC’s most important property is their ability to adhere to and invade mucosal cells [13]; an interaction with immune cells (and especially macrophages) is less frequent. Again, both these phenotypic features are heterogeneous and vary depending on both the strain’s clonality and host determinants [26]. For a strain to be assigned as an AIEC, an in vitro assay with epithelial cells is used; at least 0.1% of the bacteria in the initial inoculum must enter the cells. However, this ratio depends strongly on the cell line used in the assay (e.g., Int-407 or Caco-2) [27]. Furthermore, different AIEC clones can show different levels of cell invasion in the same assay [12]. Although these in vitro assays can discriminate between AIEC strains and non-AIEC strains isolated from healthy individuals, patients with IBD, and patients with other pathologies, we reasoned that additional in vitro models are required to study AIEC virulence and characterize the bacteria’s detrimental impact on intestinal homeostasis in the host.

The soil-living nematode worm *Caenorhabditis elegans* is a simple, powerful model of host-bacteria interactions. Many of its biological pathways are conserved in higher organisms [28], and *C. elegans* models have been used to study bacterial pathogenesis [29] and identify beneficial microorganisms [30]. The association between *C. elegans* and the bacteria found within the nematode is more than a simple predator–prey dietary relationship. In fact, several important features of *C. elegans’* innate immune system are conserved in vertebrates. Similarly, many of the virulence factors used by bacterial pathogens to cause disease in mammalian hosts are also crucial for pathogenesis in *C. elegans*. The *C. elegans* model has been used to study major human pathogens, including entero-invasive *Salmonella enterica* [31,32,33], certain enteropathogenic *E. coli* pathotypes [34,35,36], and uropathogenic *E. coli* (UPEC) [37] (which share features with AIEC [38]). To date, the LF82 prototype is the only AIEC strain to have been evaluated in the *C. elegans* infection model. When compared with the auxotrophic OP50 *E. coli* strain (*C. elegans*’ usual food in the lab), exposure to live LF82 bacteria markedly reduced the nematode’s median lifespan (LT_50_) by nearly 50% [39]. In contrast, heat-killed bacteria had no impact on survival [39]. To the best of our knowledge, no other AIEC isolates (and especially clones covering the spectrum of virulence in epithelial cell cultures) have previously been assessed for their effect on *C. elegans* longevity. In the present study, we therefore thought to determine whether the characteristics of *C. elegans* infection by different AIEC clinical isolates were related to the strains’ pathogenic potential.

## 2. Materials and Methods

### 2.1. Chemicals and Reagents

All chemicals and reagents were purchased from Sigma Aldrich Chimie (St Quentin Fallavier, France), unless otherwise stated.

### 2.2. Bacterial Strains and Culture Conditions

The *E. coli* strains included reference strains known to be invasive (or not) and clinical isolates. The strains were cultured in Luria–Bertani (LB) medium at 30 °C or 37 °C, with shaking. The uracil auxotroph OP50 is the “gold-standard” control strain routinely used in *C. elegans* survival assays [40]. OP50 has a low metabolic rate, which limits bacterial proliferation within the gut and thus maximizes the worm’s life span [41]. MG1655 is a prototype non-invasive, non-pathogenic, human-commensal-derived *E. coli* K-12 strain used as a model in bacterial genetics, molecular biology, and biotechnology [42]. *E. coli* Nissle 1917 (EcN, also known as DSM 6601 or Mutaflor) is a non-invasive, non-pathogenic, commensal strain originally isolated from the stools of a World War I soldier withstanding a severe shigellosis outbreak; the strain has many anti-infectious and anti-inflammatory probiotic properties [43,44]. The LF82 strain is the most extensively studied AIEC and is considered to be the reference strain for the association with CD [6]. Like LF82, strain EC-6362 was isolated from ileal lesions of patients with CD, and has strong invasive capacities [11]. NRG857c is also a prototype AIEC strain with additional intestinal profibrogenic traits [45,46]. The other strains used in the present study were human non-AIEC or AIEC isolates from the University of Lille’s collection (Lille, France) [47,48]: EC-6029, EC-6089, EC-6097, EC-6100, EC-6259, EC-7033, EC-7074, EC-7090, EC-7101, EC-7103, EC-7033, EC-7107, EC-7113, and EC-7137. All patients had given their written, informed consent to the isolation of bacterial strains from their biopsies or stools. The collection’s constitution had been approved by an institutional review board (references: CCPPRB Lille 1994 #94/01 and CCPPRB Lille 2000 #00/60). Of note, all bacterial strains were confirmed to be susceptible to gentamicin to ensure the appropriate use of this antimicrobial for invasion assays (data not shown).

### 2.3. C. elegans Strains, Culture Conditions, and Longevity Assays

The wild-type *C. elegans* Bristol N2 strain was provided by the *Caenorhabditis* Genetics Center (Minneapolis, MN, USA). *C. elegans* strain PX627 [49] was obtained from the CGC (kindly via Jonathan Ewbank). Worms were maintained at 20 °C or 25 °C on nematode growth medium (NGM) agar with *E*. *coli* OP50 as a food source, using established procedures [40]. Infection assays were performed on NGM plates in triplicate at least. For each bacterial strain, more than 75 worms were used in the longevity assay.

To measure the lifespan of *C. elegans* N2, synchronized L1 larvae were grown at 20 °C until they reached the L4 stage. The worms were incubated at 25 °C on NGM plates containing 5-fluoro-2′-deoxyuridine (FUdR, 50 μM). and seeded with *E. coli* (OP50 as a control, or the other strains investigated here) with 10 μL (OD = 2.0) for each lawn and incubated at 25 °C. Live or dead worms were counted every 24 h; a worm was considered to be dead when it failed to respond to a gentle touch with a worm picker. Worms that crawled off the plates or died from non-natural causes (such as bagging or adherence to the wall of the plate) were censored. All longevity assays were performed in triplicate at least with 25–35 worms per plate; hence, between 75 and 100 worms per bacterial species were used.

*C. elegans* strain PX627 was generated recently to allow auxin-mediated sterility induction [49] and is a very useful strain for worm aging studies. Auxin induces sterility in PX627 worms and thus avoids a mixture of generations in killing assays. Auxin-induced self-sterility is comparable to FUdR-induced sterility but is non-toxic and avoids the compound interactions with other experimental treatments. In fact, FUdR significantly increases lifespan, health parameters, and mitochondrial function, relative to auxin-treated PX627 worms and nontreated controls [50]. To isolate eggs and synchronize worm populations, gravid adults were bleached using standard laboratory procedures. The eggs were incubated on food-free NGM plates overnight at 25 °C. L1 larvae were then transferred to NGM plates containing 1 mM indole 3 acetic acid and seeded with *E. coli* OP50. Plates were incubated at 25 °C for about two days until worms reached the young adult stage. For longevity assays with *C. elegans* strain PX627, 35 mm NGM plates (five per *E. coli* strains) were inoculated with a drop of an overnight bacterial culture and incubated overnight at 30 °C. Plates were allowed to cool to room temperature before seeding with young adult hermaphrodite PX627 worms (20 per plate). The plates were then incubated at 25 °C and scored daily for live worms for about two weeks. Alternatively, a worm was considered to be dead and was removed when it no longer responded to being touched with a platinum wire. Worms that died as a result of becoming stuck to the plate wall were censored.

All longevity assays were performed at least twice.

### 2.4. Cell Lines, Cell Culture, and Epithelial Cell Invasion Assays

The human embryonic ileum intestine-407 (I-407) cell line (ATCC CCL6, Manassas, VA, USA) was derived from human embryonic ileum. The cells were cultured at 37° in an atmosphere containing 5% CO_2_ C in Basal Medium Eagle (ThermoFisher, llkirch, France) supplemented with 10% (*v*/*v*) heat-inactivated fetal calf serum (Eurobio Scientific, Les Ulis, France), 1% L-glutamine (ThermoFisher, Illkirch, France), 100,000 U/L penicillin, and 100 mg/L streptomycin (ThermoFisher, llkirch, France). The cell invasion assays were based on a gentamicin protection method, as described elsewhere [7]. Briefly, 24-well plates containing 4 × 10^5^ cells/well were incubated for 20 h in the absence of antibiotics and then infected for 3 h with the *E. coli* strains, at a multiplicity of infection (MOI) of 10. The cells were washed twice with PBS, and extracellular bacteria were killed by adding fresh cell culture medium containing 100 μg/mL of gentamicin for 1 h. After three washes with PBS, the cells were lysed by adding 1 mL of 1% Triton X-100 to each well for 5 min. The number of previously intracellular bacteria was determined by plating. The invasion ratio (the number of intracellular *E. coli* divided by the total number of *E. coli* in the initial inoculum) was multiplied by 100 and thus expressed as a percentage. An isolate was considered to be invasive when the invasion ratio was 0.1% or more. Each experiment was performed in triplicate. Non-invasive *E. coli* strains (K12 C600, MG1655, and EcN) and invasive *E. coli* strains (AIEC LF82 and AIEC NRG857c) were used respectively as negative and positive controls in each experiment. The invasion ratio data were quoted as the mean ± standard deviation.

### 2.5. Microscopy

Confocal microscopy was performed using a Carl Zeiss LSM 780 microscope equipped with five lasers (405, 458, 488, 514, and 633 nm). Fluorescent labeling of lectins was done according to the general principles of lectin chemistry and bioconjugate techniques. Epithelial cells were infected according to the gentamicin procedure method with 10 bacteria per cell and incubated at 37 °C under 5% CO_2_. After 3 h of incubation, host cells were washed twice with DPBS, fixed in 4% paraformaldehyde, and further stained for microscopic analysis. Host cell membranes were stained in red using fluorescent wheat germ agglutinin (WGA-Alexa 594, Vector Laboratories, Burlingame, CA, USA) at a final concentration of 5 μg/mL whereas nuclei are stained using 1 μM DAPI (Sigma-Aldrich, Saint Louis, MO, USA). Bacteria were stained in green using a primary rabbit anti-LPS polyclonal antibody (1:5000) and a secondary rabbit anti-rabbit Alexa Fluor-488-conjugated antibody (1:5000), (ThermoFisher, Illkirch, France).

### 2.6. Statistical Analysis

Interstrain differences in epithelial cell invasion were probed using a non-parametric, one-way analysis of variance (the Mann–Whitney U test). Nematode survival was analyzed using the Kaplan–Maier method; differences between survival curves were assessed in a log rank (Mantel Cox) test and (for borderline results) the Gehan–Breslow–Wilcoxon test in Prism software (GraphPad Software, San Diego, CA, USA). Associations between variables were qualified by the *p*-value-assigned Spearman rank correlation coefficient, together with linear (Pearson) regression using the XLSTAT add-on (Addinsoft, Bordeaux, France) for Excel^®^ (Microsoft, Inc., Redmond, WA, USA). The threshold for statistical significance was set to *p* < 0.05.

## 3. Results

### 3.1. Strain-Specific E. coli Infection Impacts the Survival of C. elegans

**Figure 1:**
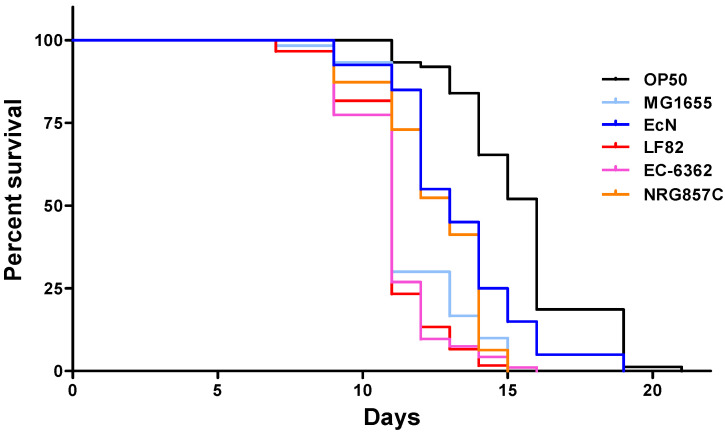
Kaplan–Meier survival plots of killing assays for *C. elegans* (N2) infected with six different *E. coli* strains. The “*n*” values in brackets correspond to the initial number of larvae investigated for each bacterial strain. L4 larvae were transferred to lawns of OP50 (the control strain: *n* = 84), MG1655 (*n* = 74), EcN (*n* = 45), LF82 (*n* = 78), EC-6362 (*n* = 101), and NRG857C (*n* = 65).

**Figure 2:**
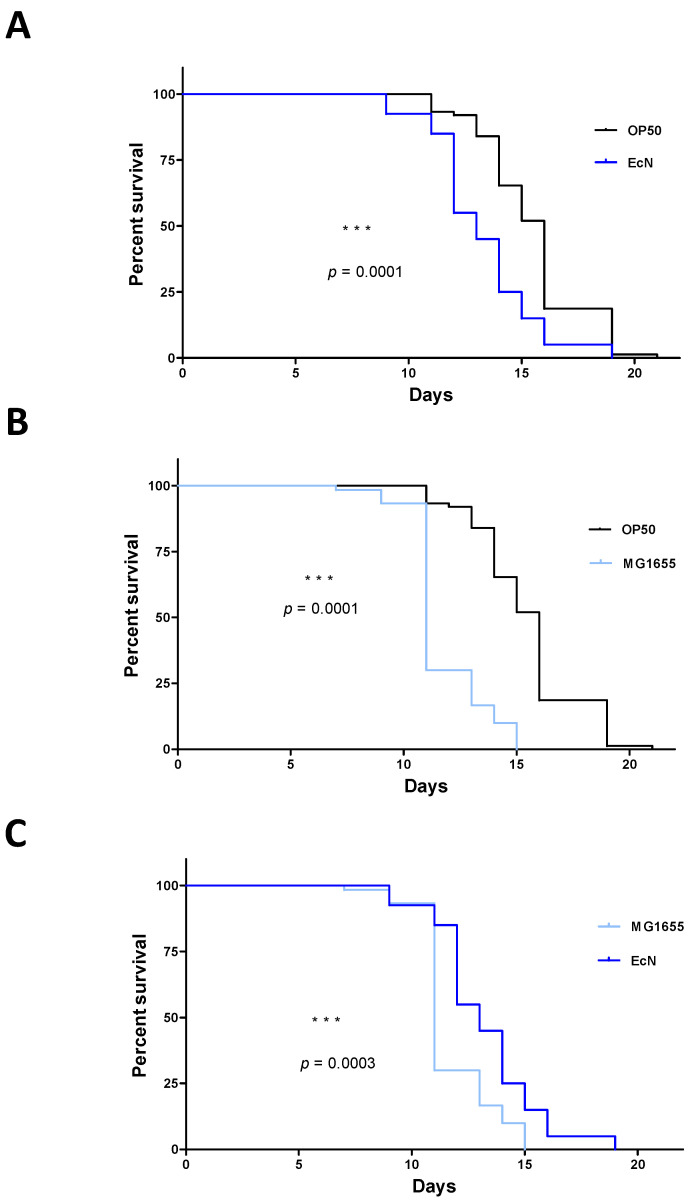
Pairwise comparisons of the effects of *E. coli* strains on *C. elegans*’ lifespan. The “*n*” values in brackets correspond to the initial number of larvae investigated for each bacterial strain (**A**) OP50 (*n* = 84) vs. EcN (*n* = 45). (**B**) OP50 (*n* = 84) vs. MG1655 (*n* = 74). (**C**) MG1655 (*n* = 74) vs. EcN (*n* = 45). The statistical significance of intergroup differences was assessed with a log-rank (Mantel-Cox) test; ***: *p* < 0.001.

**Figure 3:**
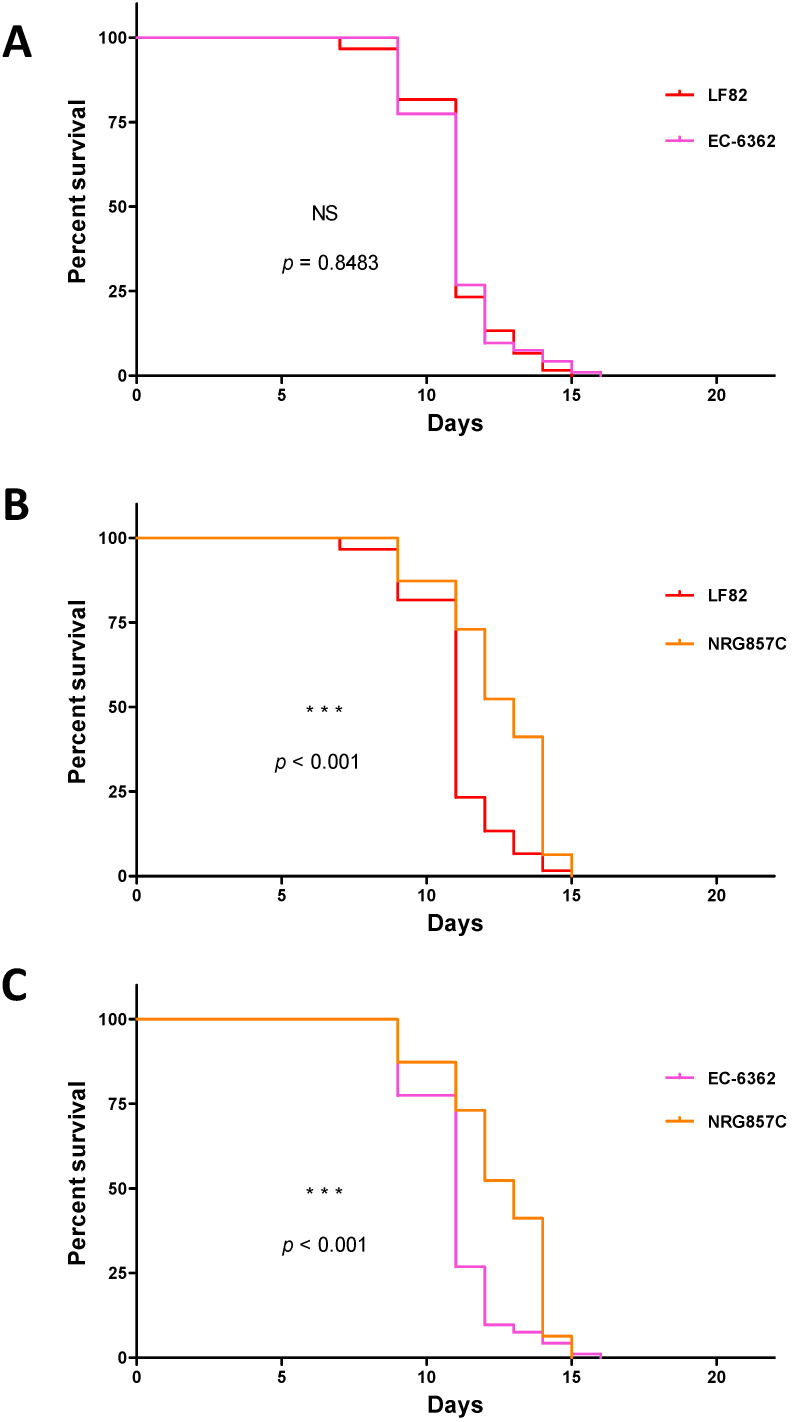
Pairwise comparisons of *C. elegans* lifespan-modulating effects of *E. coli* strains. The “*n*” values in brackets correspond to the initial number of larvae investigated for each bacterial strain (**A**) LF82 (*n* = 78) vs. EC-6362 (*n* = 101). (**B**) LF82 (*n* = 78) vs. NRG857c (*n* = 65). (**C**) EC-6362 (*n* = 101) vs. NRG857c (*n* = 65). The statistical significance of intergroup differences was assessed with a log-rank (Mantel-Cox) test; ***: *p* < 0.001; NS: not significant).

**Figure 4:**
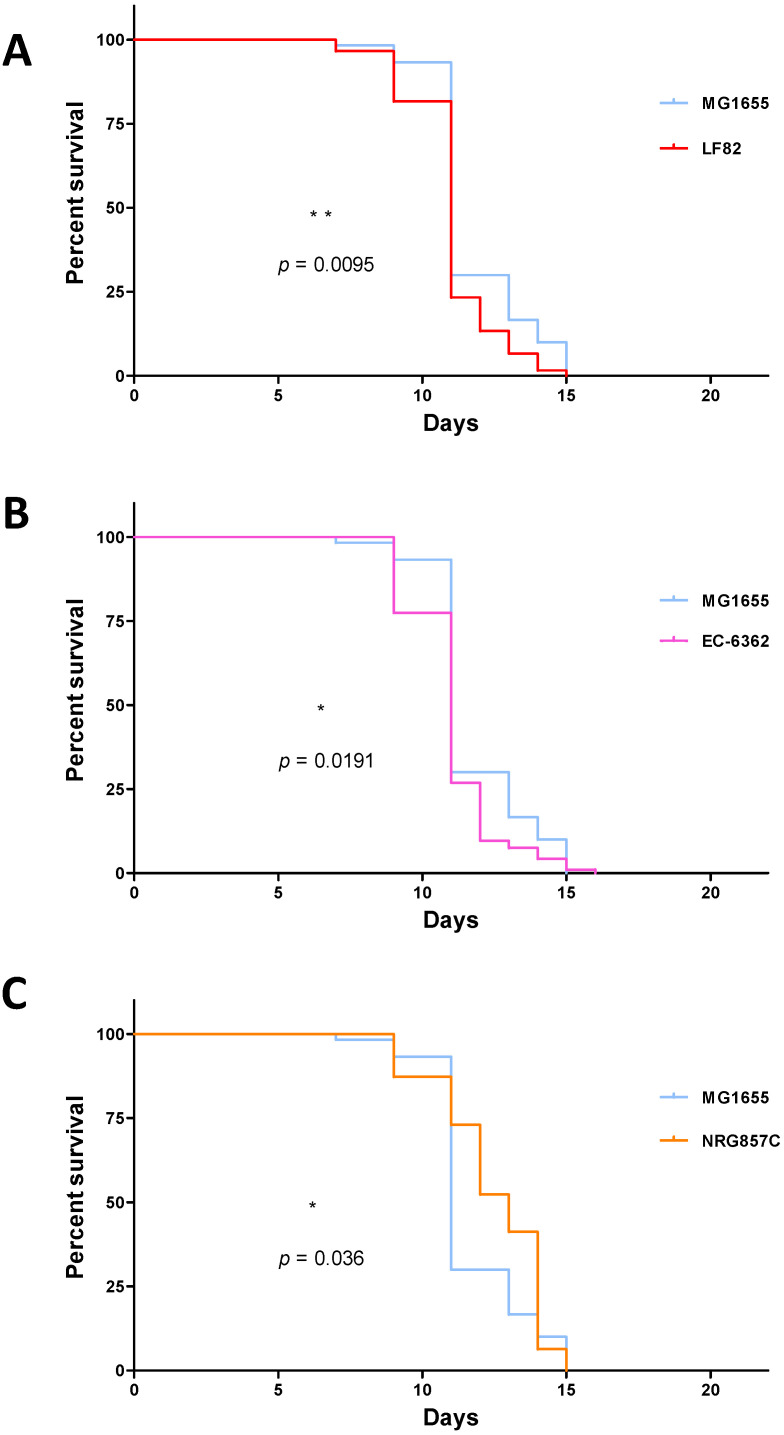
Pairwise comparisons of *C. elegans* lifespan-modulating effects of *E. coli* strains (**A**) MG1655 (*n* = 74) vs. LF82 (*n* = 78). (**B**) MG1655 (*n* = 74) vs. EC-6362 (*n* = 101). (**C**) MG1655 (*n* = 74) vs. NRG857c (*n* = 65). The “*n*” values in brackets correspond to the initial number of larvae investigated for each bacterial strain. The statistical significance of intergroup differences was assessed with a log-rank (Mantel-Cox) test; *: *p* < 0.05; **: *p* < 0.01).

In a first set of experiments, we evaluated *C. elegans*’ lifespan upon infection with three non-invasive non-pathogenic *E. coli* strains (OP50, MG1655, and the probiotic-like EcN), and three AIEC strains (LF82, EC-06362, and NRG857c) (Figure 1). N2 worms feeding on OP50 showed the greatest longevity, with an LT_50_ of 16 days. In contrast, *C. elegans*’ LT_50_ was shortened to 11 to 13 days by the other bacterial strains assessed. There were significant differences between OP50 on one hand and the non-invasive EcN and MG1655 strains on the other (*p* < 0.01 in a log-rank test, Figure 2A,B). Furthermore, LT_50_ was significantly shorter for MG1655 than for EcN (Figure 2C). When considering strains with invasive properties, LT_50_ was 11 days for LF82 and EC-6362 and 13 days for NRG857c. Whereas the *C. elegans* survival curves for the AIEC strains LF82 and EC-6362 were essentially superimposable (Figure 3A), the lifespans was significantly longer for the non-invasive MG1655 strain (*p* < 0.01 in a log-rank test, Figure 4A,B) and the AIEC NRG857c (*p* < 0.01, Figure 3A,B). Unexpectedly, *C. elegans*’ lifespan was longer for the profibrogenic pathobiont NRG857c than for the non-invasive MG1655 (Figure 4C).

Of all the strains investigated here, the anti-inflammatory EcN was associated with the longest lifespan (with the exception of OP50, whose low proliferation rate in the gut maximizes *C. elegans*’ lifespan), when compared with the AIEC NRG857c (*p* = 0.0327, data not shown). Overall, the AIEC phenotype did not appear to be tightly associated with a shortening of *C. elegans*’ lifespan.

### 3.2. AIEC Strains Differ in Their Cell Invasion Properties In Vitro

**Figure 5:**
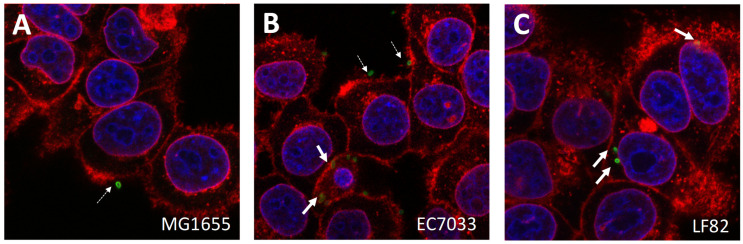
Confocal microscopy illustrating the rare events of cell-adhering (extracellular) and cell-invaded (intracellular) *E. coli* strains on I-407 cells after a 3 h incubation and extensive washings. (**A**): Non-invasive MG1655 (**B**): EC.7033 AIEC. (**C**): LF82 AIEC. Extracellular (dotted arrows) and intracellular (bold arrows) bacteria are identified by immune detection (green); Epithelial cells are contrasted by DAPI-labelled nuclei (blue) and WGA-labelled membranes (red) (see methods). Original magnification ×63.

**Figure 6:**
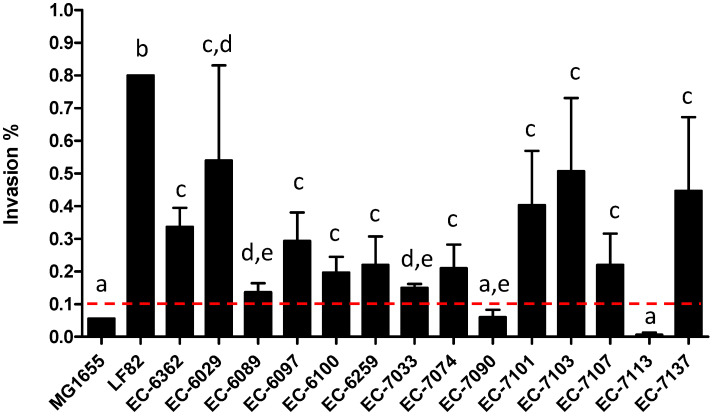
Strains of *E. coli* (*n* = 16) different in their ability to invade I-407 cells. The invasion ratio (the number of intracellular *E. coli* divided by the total number of *E. coli* in the initial inoculum at a MOI of 10) after a 3 h incubation and a 1 h treatment with gentamicin was multiplied by 100 and thus expressed as a percentage. Strains with an invasion index ≥0.1% (the threshold indicated by a dotted line) are classified as AIEC. The data are quoted as the mean ± standard deviation from three determinations. The various letters indicate statistically significant differences (*p* < 0.05) between strains in a one-way analysis of variance (the Mann–Whitney U test).

By definition, an AIEC must have an invasion ratio of 0.1% of the initial inoculum at a MOI of 10 on I-407 epithelial cells [7]. Given this somewhat arbitrary threshold, individual strains can exhibit different levels of invasiveness and/or borderline invasiveness. These strain-specific properties were highlighted by confocal microscopy of stained intracellular *E. coli* (Figure 5). We performed invasion assays with 16 *E. coli* human isolates selected as putative AIEC, together with MG1655 and LF82 (Figure 6). The degree of invasiveness ranged from non-invasive (invasion ratio <0.1%) for MG1655, EC-7090, and 7113 to moderate (invasion ratio between 0.1 and 0.25%) for EC-6089, EC-6100, EC-6259, EC-6029, EC-7033, EC-7074, and EC-7107 and highly invasive (invasion ratio >0.25%) for LF82, EC-6362, EC-6097, EC-7101, EC-7103, and EC-713. These data confirmed that as an intrinsic phenotypic characteristic, individual AIEC strains differ in their invasiveness. 

### 3.3. Assessment of the *AIEC’s Pathogenicity in* C. elegans

**Figure 7:**
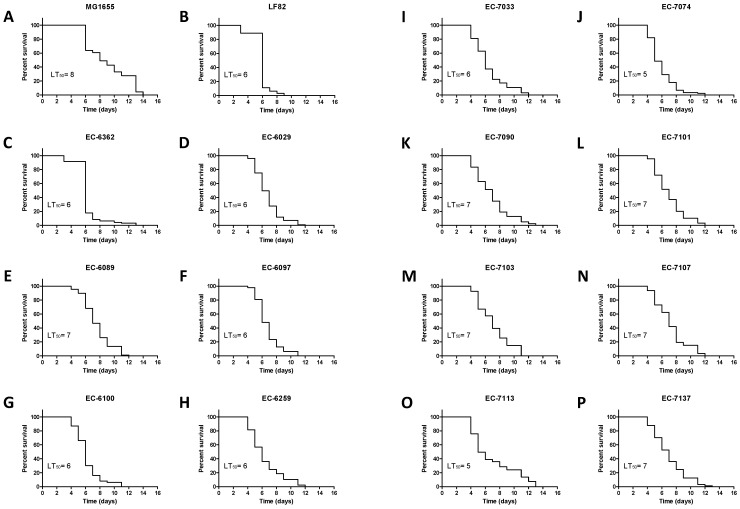
Kaplan–Meier survival plots from individual killing assays for *C. elegans* (PX627) infected with 16 distinct *E. coli* strains. The “*n*” values in brackets correspond to the initial number of larvae investigated for each bacterial strain. *C. elegans* young adults were transferred to lawns of (**A**) MG1655 (*n* = 94), (**B**) LF82 (*n* = 98), (**C**) EC-6362 (*n* = 96), (**D**) EC-6029 (*n* = 101), (**E**) EC-6089 (*n* = 88), (**F**) EC-6097 (*n* = 94), (**G**) EC-6100 (*n* = 100), (**H**) EC-6259 (*n* = 97), (**I**) EC-7033 (*n* = 94), (**J**) EC-7074 (*n* = 89), (**K**) EC-7090 (*n* = 78), (**L**) EC-7101 (*n* = 89), (**M**) EC-7103 (*n* = 94), (**N**) EC-7107 (*n* = 93), (**O**) EC-7113 (*n* = 99), and (**P**) EC-7137 (*n* = 97). The median lifespan (LT_50_) for each evaluated strain is expressed in days.

**Figure 8:**
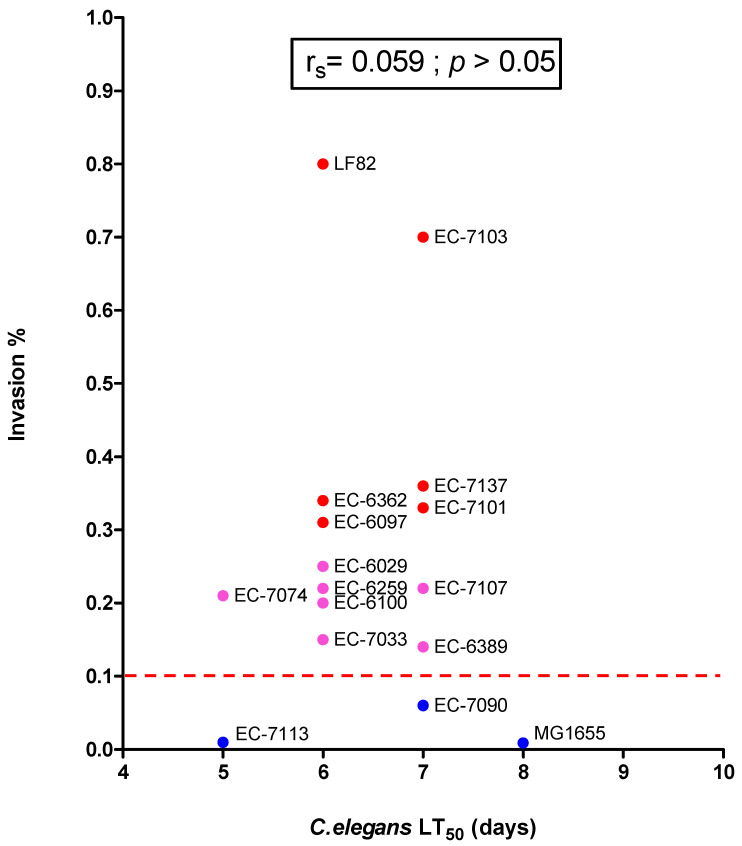
Lack of association between the median lifespan (LT_50_) of *E. coli*-infected *C. elegans* and the corresponding invasion ratio with the I-407 epithelial cell line, for 16 distinct *E. coli* strains. Strains with an invasion index ≥0.1% (the threshold indicated by a dotted line) are classified as AIEC. The correlation’s statistical significance was assessed by calculating Pearson’s regression coefficient and Spearman’s non-parametric rank correlation r_s_ coefficient.

In order to mitigate possible bias due to exposure to FUdR in the longevity assay, we used the PX627 *C. elegans* strain. Auxin treatment restricted to the L1 to L4 development phase is suffisant to induce PX627 sterility (see the Methods section) and thus induces sterility. We assessed survival upon growth on three non-invasive *E. coli* strains (*n* = 3), seven moderately invasive strains, and six highly invasive strains, (Figure 7A–P). LT_50_ ranged from five days for the non-invasive EC-7113 strain to eight days for MG1655. However, the invasion ratio in an in vitro cell culture assay was not correlated with virulence in the *C. elegans* longevity assay. For instance, a moderately invasive strain (EC-7074) had a short LT_50_ (5 days), while a highly invasive strain (EC-7103) had an LT_50_ of seven days. Our data confirmed that clinical *E. coli* isolates differ in their impact on *C. elegans*’ longevity. Some of the highly invasive strains lengthened the lifespan, and the correlation between the invasion ratio and LT_50_ (as assessed with Pearson’s coefficient (r^2^ = 8.9 × 10^−6^) and Spearman’s non-parametric rank correlation coefficient (rs = 0.059)) was not statistically significant (Figure 8). Hence, the AIEC’s invasiveness was clearly not correlated with *C. elegans*’ lifespan.

## 4. Discussion

Identification of the AIEC pathotype is still challenging; most of the techniques currently used to investigate host–pathobiont cross-talk are based on the infection of (immortalized) cell lines [24]. These assays are sometimes limited by the cell lines’ loss of key physiological features. Hence, integrated and/or more relevant models are required. The use of human intestinal cells derived from a patient’s colonic organoids has deepened our understanding of the physiopathology of IBD, inter-individual variability, and the lesions’ precise location in the gut [51]. Although the organoid model offers many advantages, it is still time-consuming and costly and requires the patient’s consent for access to biopsies. Preclinical animal models (e.g., such as chemically-induced inflammation in rodents [11,52,53] or susceptible transgenic mice [52]) are useful for identifying specific pathogenic effects of AIEC on the host (such as the induction of fibrogenesis [54] and autophagy [55,56]), or highlighting the role of the microenvironment [57,58] and bacterial virulence factors [59,60]. However, these animal models are not practical for extensive screening of a large number of strains nor for extended therapeutic approaches [8].

As a simple host that multiplies quickly, *C. elegans* has emerged as a robust animal model for investigating microbial pathogenesis [61,62] and the beneficial effects of certain bacteria [30]. In the scientific literature, there are a few studies of the virulence of a small number of strains from the same bacterial species. Strain specificity has been clearly linked to levels of virulence for Gram-positive *Staphylococcus aureus* [63] and Gram-negative *Pseudomonas aeruginosa* and *Klebsiella pneumoniae* [64]. Interestingly, the most pathogenic strains did not show the highest level of colonization. With regard to *E. coli*, most of the studies concerned enterohemorrhagic, enteropathogenic, and enterotoxinogenic pathovar strains that kill *C. elegans* in a few hours [35]. In contrast, commensal bacteria (including pathobionts lacking known invasive determinant genes) have been rarely studied. Simonsen et al. [39] showed that live LF82 AIEC (but not heat-killed counterparts) drastically shortened *C. elegans*’ lifespan, relative to the OP50 strain. Moreover, several LF82 mutants were found to be attenuated in the killing assay, which suggested that several *E. coli* genes were associated with loss of tolerance and mortality [39]. In a study of the MG1655 K-12 strain (lacking the O antigen), recombinant derivatives producing various O antigens were considerably more virulent in the lifespan assay [65]. Furthermore, the commensal *E. coli* HS strain [66] killed *C. elegans* as efficiently as an enteroaggregative strain or the O antigen producing strains. The results suggest that neither commensal *versus* pathogenic status nor a strain’s origin accurately predicts the effect on *C. elegans* lifespan. Accordingly, the results of infection assays with various well-characterized extended-spectrum-β-lactamase-producing *E. coli* and β-lactam-susceptible *E. coli* isolates indicated that the ability to kill *C. elegans* was correlated with the presence of virulence factors, and that CTX-M-producing isolates had a relatively low impact on the nematode [67]. Schifano et al.’s study of UPEC from different phylogroups showed that the ability to adhere to and invade epithelial cells was not correlated with shorter lifespan nor with gut colonization in the nematode model, although pathogenicity was more closely related to the level of oxidative stress [37]. Our present results are in line with the literature data, since AIEC strains are similar to extra-intestinal pathogenic *E. coli* strains and UPEC strains with regard to their ability to adhere and invade host cells [38,68]. 

Overall, the worm’s responses to bacterial infections might partly be influenced by (i) the *C. elegans* strain used and (ii) the method to induce sterility and prevent progeny arising during the assay. For example, the OP50-mediated LT_50_ was determined to be 6 days or 10 days in two studies using the Bristol N2 strain on FUdR plates or daily transfer onto new plates, respectively [65,69]. However, the OP50-mediated LT_50_ was 12 to 14 days in experiments with the SS104 temperature-sensitive (25 °C) *C. elegans* mutant [39]. Here, we confirmed that the lifespan of PX627 worms was shorter with the LF82 AIEC prototype than with OP50 [39]. Use of the PX627 *C. elegans* strain enabled us to avoid sterilizing treatment with FUdR—a compound that might affect the worm and the bacterium. Indeed, FUdR has antineoplastic properties and might inhibit cell invasion by AIEC, as has been demonstrated for the analogous drug 6-mercaptopurine [70].

As mentioned above, the adhesion and invasiveness of the AIEC strains are assessed with human epithelial cells. However, these properties are cell-line dependent. Variations in the invasion ratio and survival inside macrophages have also been reported [12,26]. Furthermore, a given strain’s invasiveness is also influenced by environmental parameters such as the culture medium [71], bacterial catabolites [72], bacterial metabolites (e.g., propionate and short chain fatty acids [73,74]), other host-derived molecules (e.g., lactoferrin [75]), and emulsifiers present in food [58,76]. Taken as a whole, variable and potentially unknown determinants of AIEC status and virulence might account for the inconsistent nature of the AIEC’s interaction with cell lines and the lack of correlation with *C. elegans*’ lifespan. Another limitation is linked to the incubation temperature for the *C. elegans* invertebrate model (25 °C), which differs markedly from the host (human) body temperature.

In conclusion, the intrinsic invasiveness of AIEC with intestinal epithelial cell line (i.e., the fact for an individual strain to be characterized as invasive or not together with the intensity of invasion, established in % from the infectious inoculum), was not correlated with *C. elegans*’ lifespan. Provided that the worm’s lifespan is low when incubated with certain AIEC strains (such as LF82), this model could nevertheless be used to screen drugs or anti-invasive probiotic-like strains for their ability to increase/restore longevity in *C. elegans*. This strategy could be used to target specific AIEC clones via competition [69,77,78,79] or via more general anti-inflammatory and anti-oxidant properties [80]. Indeed, inoculation with certain probiotic bacteria (including strains of *Propionibacterium freundenreichii*, bifidobacteria spp., and many lactobacilli spp.) is associated with greater *C. elegans* survival in a strain-specific manner [30,81,82]. Interestingly, bacteria with intrinsic anti-inflammatory and anti-oxidant properties extended the lifespan of *C. elegans* and were also able to alleviate colitis in mice [83].

## Data Availability

The data presented in this study are available on request from the corresponding author.
